# Cytopathic changes in mpox and herpesvirus infections: Role of histopathology and Tzanck smear

**DOI:** 10.1016/j.jdcr.2024.08.023

**Published:** 2024-09-21

**Authors:** Francisco José Rodríguez-Cuadrado, Cristian Fernando Caballero-Linares

**Affiliations:** Department of Dermatology, Hospital Universitario Puerta de Hierro Majadahonda, Majadahonda, Madrid, Spain

**Keywords:** dermatopathology, herpesvirus, histopathology, mpox, Tzanck, viral infections

*To the Editor:* We have carefully read the article published by Ravichandran et al,^1^ which describes the case of an HIV-negative male in whom there was a diagnostic doubt between herpesvirus infection and mpox infection. We are pleased to see that the multicenter study we publish has provided a reference for the indication of a biopsy in doubtful cases.^2^

There is not much literature regarding Tzanck test evaluation of mpox lesions. In a case recently published by Derrick et al,^3^ the Tzanck smear showed no specific cytologic alterations, as no inclusion bodies or multinucleated giant cells were present. However, biopsy showed multinucleated keratinocytes and necrosis of both epidermis and pilosebaceous unit. It is interesting how the Tzanck test was negative even in the presence of concomitant herpes simplex virus 1 infection. This may be because of the limited sensitivity of this test in late lesions or pustules.^4^

In contrast, in the case presented by Ravichandran et al,^1^ the Tzanck test did reveal multinucleated giant cells. As reported, inclusion viral bodies could be observed in the biopsy.

It is noteworthy to comment on 2 major differences in the histology of skin changes resulting from herpesvirus infection compared with those caused by mpox. First, although inclusion bodies can be observed in both infections, those of mpox are located intracytoplasmic (Guarnieri eosinophilic inclusion bodies), whereas those of herpesvirus are intranuclear. Second, the changes occurring at the nuclei are different: herpes-infected keratinocytes present a nucleus with a “steel gray” appearance and chromatin margination, whereas the nuclei of mpox-infected keratinocytes sometimes present a “ground glass” appearance, although this is an inconstant finding.^2^^,^^5^

It will be of interest to further investigate whether the Tzanck test could present an acceptable sensitivity in the detection of such changes, potentially serving as a less invasive diagnostic alternative to the biopsy, and providing faster results than that obtained by polymerase chain reaction.

## Conflicts of interest

None disclosed.
